# The role of the North American continent in strengthening the Asian summer monsoon

**DOI:** 10.1126/sciadv.adu8589

**Published:** 2025-09-05

**Authors:** Linlin Chen, Paul J. Valdes, Alexander Farnsworth

**Affiliations:** ^1^ School of Geographical Sciences and Cabot Institute, University of Bristol, Bristol, BS8 1SS, UK.; ^2^ State Key Laboratory of Tibetan Plateau Earth System, Environment and Resources (TPESER), Institute of Tibetan Plateau Research, Chinese Academy of Sciences, Beijing, China.

## Abstract

Most studies on modern Asian monsoon formation focus on mechanisms arising within the Afro-Eurasian continent, while fewer compare the effects from remote continents. Here, we explore this question using a coupled climate model. We show that the existence of the North American continent is critical for the intensity of the Asian summer monsoon. The mechanism involves North America acting as an additional heating center, resulting in the strengthening and extension of oceanic advection toward the monsoon region. This is achieved via the Rodwell-Hoskins mechanism that strengthens the North Pacific subtropical high and through a widespread Northern Hemisphere heating that shifts the Hadly circulation subsidence center poleward. This teleconnection is independent of the presence of the Tibetan Plateau, and its impact on East Asian summer precipitation is found to be smaller but comparable to that of Tibet. The individual role of other continents outside Afro-Eurasia is found to be less important.

## Teaser

Asian monsoon precipitation is driven by the presence of the North American continent as well as Tibetan orography.

## INTRODUCTION

The Asian monsoon is one of the most important planetary climate systems, whose seasonal rains affect the lives and livelihoods of over a billion people. The high possibility of increasing extreme precipitation and lengthening monsoon season of the East Asian Summer Monsoon (EASM) and Indian Summer Monsoon (ISM) over the coming century underscores the need to understand its driving processes, mechanisms and variability (*1*).

The Asian monsoon is affected by factors on various timescales. In the Quaternary [2.58 to 0 million years ago (Ma)], changes in the Asian monsoon are strongly linked to variations in Earth’s orbit, as well as associated changes in greenhouse gases and ice sheets (*2*–*4*). When sea levels dropped due to ice sheet expansion in the Quaternary, more land area of the Maritime Continent became exposed, which has been shown to influence the Asian monsoon (*5*). Yet, the relative role of the areal extent of Antarctica and other landmasses has seen little investigation.

On longer geological timescales before the Quaternary, the role of changing continental positions and topography becomes more important (*6*). Most studies on the mechanisms controlling the formation of the modern Asian monsoon focus on local aspects within the Afro-Eurasian continent, particularly land-sea distribution and orography (*7*–*10*), especially in the Tibetan-Himalayan region (*6*, *11*–*13*). Recent studies have also considered teleconnections from the Australian continent to the Asian monsoon dynamics. For example, Yin and Nie (*14*) demonstrated that the latitudinal location of Australia can influence EASM onset through perturbing cross-equatorial flow.

However, the roles of the North American and South American continents in Asian monsoon formation have been largely overlooked. Some studies indicate the linkage between them. Modern observations, paleoclimate records, and climate simulations of the Last Glacial Maximum, present-day, and future scenarios all suggest the existence of an Asia-Pacific-North America teleconnection across intraseasonal to millennial timescales, often characterized by contrasting wet and dry patterns between Asia and North America (*15*–*19*). However, most of these studies examine how the Asian monsoon influences remote climate systems, with fewer exploring the “reverse” relationship of how changes in the Americas might influence Asia. Evidence from geological history hints at such a connection. For example, the closure of the Panama gateway at around 5 Ma led to a marked increase in ISM rainfall (*20*). More directly, He *et al.* (*21*) showed that the westward drift of the North American continent and the Rocky Mountains from the Eocene (~55 Ma) to the present day contributed to a southward contraction of East Asian spring persistent rainfall, underscoring the potential of the American continental position in shaping Asian hydroclimate.

Few studies have directly examined the relative contributions of each unconnected continent to the Asian monsoon and none in a systematic way. One of the closest is the work of Xu *et al.* (*22*), who used an atmosphere-only general circulation model (AGCM) to incrementally add a range of continents and mountains. Their focus was on the land-sea distribution effects on zonal tropical subcontinental and meridional large scales, rather than the role of individual continents. In addition, they had to use a prescribed sea surface temperature (SST) using an AGCM, so the full impact of the continents on the Asian monsoon was not represented.

Most previous studies who explored the effects of Afro-Eurasian continent with idealized geometrics on the Asian monsoon used AGCMs, requiring prescribed SSTs, either as a zonal mean distribution with maxima at the equator or using present-day SST, that eliminate potential feedbacks from changing land-sea distributions on ocean circulation and ocean heat transport (*8*–*10*, *23*). A few studies alternatively used slab ocean models which do not fully resolve deep ocean dynamics and changes in ocean transport (*7*, *24*). Given that ocean circulation and the resulting SST patterns largely shape Asian monsoon development (*25*–*27*), it is therefore critical that when altering land-sea geometries, the resulting impact on the ocean is taken into account through the use of coupled atmosphere-ocean general circulation models (AOGCMs).

**Fig. 1.**
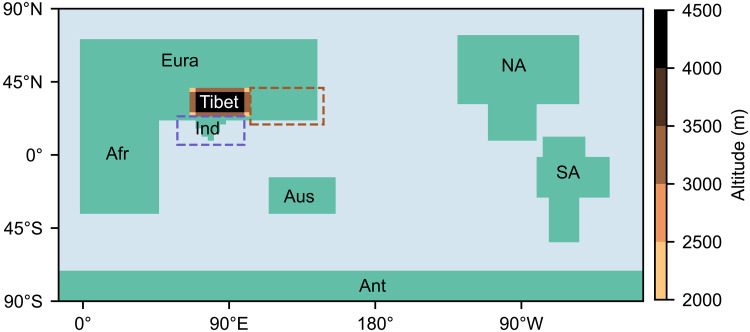
Idealized continental and orographic configuration. The abbreviations represent continents of Eurasia (Eura), India (Ind), Africa (Afr), Australia (Aus), Antarctica (Ant), North America (NA), and South America (SA). Shading indicates land-sea-mountain mask: green for land (0-m elevation), light blue for ocean, and copper for elevated terrain (Tibet, up to 4500 m). Dashed rectangles indicate analysis domains: brown for EASM region; purple for ISM region (same in the following figures).

Here, we decompose the individual impacts of each continent’s non-orographic (height set to 0 m) distribution on the modern Asian monsoon using a fully coupled AOGCM, the Hadley Centre Coupled Model (HadCM3BL), integrated to near climate equilibrium. The sensitivity experiments are conducted using different combinations of global continents. A list of experiments is provided in Table 1, and the geometries of all continents and mountains are displayed in Fig. 1 and table S1. We use idealized land-sea geometry to focus more on the presence/absence of a continent rather than the detailed geography. This simplification also enables isolation of primary large-scale responses, avoiding confounding effects from detailed geography. Previous studies have shown that flat, idealized representations of the Afro-Eurasian continent can capture key characteristics of the Asian summer monsoon (*7*–*10*), which confirms the feasibility and reliability of our experimental design. Recognizing the substantial impact of the Tibetan Plateau on the Asian monsoon, we also include an idealized Tibetan Plateau in key experiments. This allows us to assess whether certain continental effects remain in the presence of Tibet and to compare their relative importance. To separately evaluate atmospheric and oceanic contributions to a critical process, we further perform a set of atmosphere-only simulations with prescribed SSTs and sea ice. Although we use idealized continental configuration, this study advances theoretical understanding of modern Asian monsoon dynamics.

**Table 1.** Summary of experiments with varying land-sea-mountain configurations.

Experiment	Configuration description
**Atmosphere-ocean coupled experiments**	
WaterWorld	No landmass
Eura	Modern Eurasia
EuraInd	Eurasia and India
EuraIndAfr	Eurasia, India, and Africa
EuraIndAfrAus	Eurasia, India, Africa, and Australia
EuraIndAfrAnt	Eurasia, India, Africa, and Antarctica
EuraIndAfrNA	Eurasia, India, Africa, and North America
EuraIndAfrSA	Eurasia, India, Africa, and South America
ALL	All continents (Eurasia, India, Africa, Australia, Antarctica, North America, and South America)
ALL_noNA	All continents except North America
EuraIndAfr_Tibet	Eurasia, India, Africa, and Tibet
EuraIndAfrNA_Tibet	Eurasia, India, Africa, North America, and Tibet
ALL_Tibet	All continents and Tibet
EuraExtended	Eurasia extended northward to 90°N
**Atmosphere-only experiments**	
Atmos_EuraIndAfr	EuraIndAfr land-sea mask; prescribed SSTs from coupled EuraIndAfr experiment
Atmos_EuraIndAfrNA	EuraIndAfrNA land-sea mask; prescribed SSTs from coupled EuraIndAfrNA experiment
NA_LandOnly	EuraIndAfrNA land-sea mask; prescribed SSTs from coupled EuraIndAfr experiment

Model configuration and experimental design details are provided in Materials and Methods. The EASM and ISM regions are defined as 20° to 40°N, 105° to 146.25°E and 7.5° to 22.5°N, 60° to 97.5°E, respectively. There are many aspects used to describe monsoon characters. Here, we only focus on the climatological mean state of boreal summer [June-July-August-September (JJAS)] precipitation.

## RESULTS

**Fig. 2.**
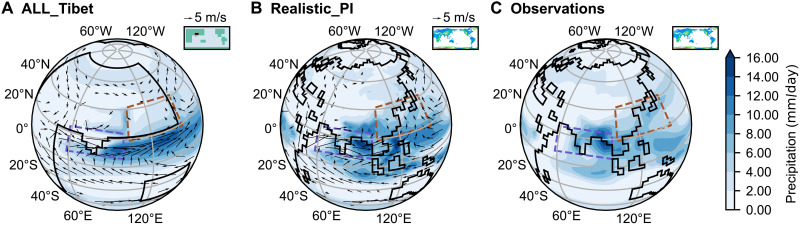
JJAS climatology for idealized, realistic, and observed configurations. JJAS precipitation (millimeters per day, shading) with horizontal wind vectors at 850 hPa (meters per second, arrows; only wind speeds >2 m/s shown) for (**A**) ALL_Tibet, (**B**) Realistic_PI, and (**C**) observations (precipitation only). Black lines indicate land-sea boundaries. Inset maps show global land-sea-mountain masks.

To assess the robustness of our simulations, we setup an idealized configuration with all flat continents and a simplified Tibetan Plateau (namely, ALL_Tibet), comparing it with a more realistic setup (Realistic_PI) that uses modern geography and preindustrial (PI) CO_2_ levels of 280 parts per million by volume (ppmv), as well as with observations. The ALL_Tibet simulation produces a mean summer climate broadly similar, although not identical, to both the Realistic_PI simulation and the observations (Fig. 2). This discrepancy is expected, given the idealized land-sea boundaries, the absence of orography outside Tibet, the lack of an Antarctic ice sheet, etc.

### Individual continental effects

The WaterWorld experiment, which has no land, produces zonally symmetric precipitation patterns, with a distinct precipitation minimum along the equator (fig. S1A). This pattern is consistent with fully coupled aquaplanet simulations with dynamic oceans (*28*–*30*) but absent in atmosphere-only aquaplanet models (*31*–*33*). The idealized modern Eurasia continent (experiment Eura) shows less area-averaged JJAS precipitation over its southern part compared to the northern part (fig. S1B). With the addition of India to Eurasia, JJAS precipitation over the Indian subcontinent decreases by 1.39 mm/day due to surface albedo changes from <0.1 over ocean to 0.3 to 0.4 over land, with negligible effects on the EASM region (fig. S1, D and E). The subsequent addition of the African continent induces minimal precipitation changes over the EASM region but enhances summer precipitation over the ISM region by 0.92 mm/day and the northern Indian Ocean (5°S to 7.5°N, 48.75° to 146.25°E) by more than 4.52 mm/day (fig. S1, F and G, and table S2). Africa disrupts low-latitude zonal easterlies and augments cross-equatorial flow over the Indian Ocean in summer (fig. S1F), resembling the modern Somali jet that traverses northward over the northern Indian Ocean.

**Fig. 3.**
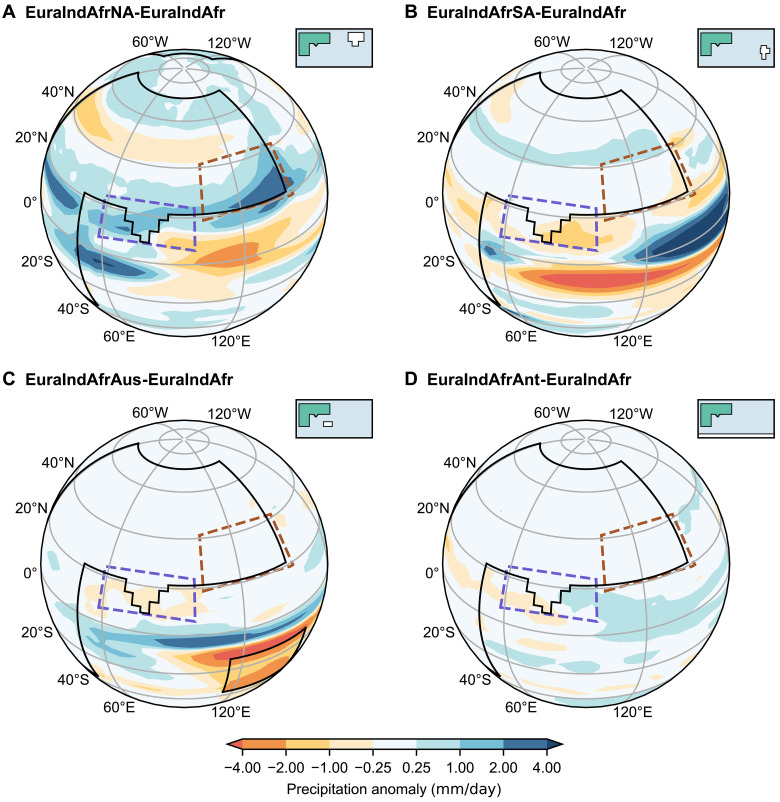
JJAS precipitation anomalies from flat-continent sensitivity experiments. Precipitation anomalies (millimeters per day, shading) show the effects of adding individual continents to the EuraIndAfr baseline: (**A**) North America (EuraIndAfrNA-EuraIndAfr), (**B**) South America (EuraIndAfrSA-EuraIndAfr), (**C**) Australia (EuraIndAfrAus-EuraIndAfr), (**D**) Antarctica (EuraIndAfrAnt-EuraIndAfr). Inset maps show global land-sea-mountain masks, where white shading highlights the landmass added in each sensitivity experiment.

Addition of the Australian, Antarctic, North American, and South American continents individually creates different precipitation responses relative to the EuraIndAfr baseline experiment (Fig. 3; fig. S1, H to O; and table S2). The largest change occurs with the addition of North America (EuraIndAfrNA experiment; Fig. 3A and fig. S1, H and I), which increases Asian summer monsoon precipitation by 1.91 mm/day, corresponding to a 23% increase relative to the ALL_Tibet configuration (percentages hereafter are relative to ALL_Tibet). This positive precipitation anomaly displays regional heterogeneity. The largest precipitation increase occurs in southeastern Asia, raising EASM precipitation by 1.28 mm/day (32%), followed by ISM precipitation by 0.63 mm/day (14%). Over the Indian subcontinent alone (excluding oceanic precipitation), the increase reaches 1.19 mm/day.

Overall, the addition of Australia, Antarctica, or South America individually has little impact on the EASM and ISM precipitation intensity (Fig. 3, B to D, and fig. S1, J to O). Australia modestly increases summer precipitation over the tropical Indian Ocean (Fig. 3C and fig. S1, L and M). South America decreases oceanic summer precipitation near southeastern India and the tropical Indian Ocean but increases precipitation over the Western Pacific Warm Pool (Fig. 3B and fig. S1, J and K). However, both the Australian and South American additions produce negligible changes to continental precipitation. Adding Antarctic continent (without an ice sheet; flat topography) has little teleconnection effects on the mean state of summer precipitation within the Asian monsoon region and the surrounding ocean (Fig. 3D and fig. S1, N and O).

Additional experiments show complex interactions and teleconnections among continents affecting Asian monsoon precipitation. When all continents are combined together (ALL; fig. S1, P and Q), the precipitation increases over the EASM region are similar to that resulting from adding only North America to EuraIndAfr configuration (fig. S1, H and I). While for ISM precipitation, there is a larger increase of 2.57 mm/day (57%) from the EuraIndAfr to ALL experiments, surpassing any single-continent addition. This implies that the addition of North America alone contributes the most to the increasing of EASM precipitation, while a strong ISM precipitation necessitates the addition of multiple remote continents acting together.

The experiment including all continents except North America (ALL_noNA) further confirms the critical role of North American interactions in enhancing Asian summer precipitation. When North America is excluded, precipitation changes over the Asian summer monsoon region relative to the EuraIndAfr experiment are minor, and precipitation even decreases over the ocean region south of Eurasia (fig. S1, R and S). Overall, these simulations provide robust evidence that North American teleconnection plays an essential role in shaping modern EASM and ISM precipitation.

### Atmospheric and oceanic contributions to North America–Asia teleconnection

To evaluate the relative roles of North America’s landmass and its SST influence on Asian summer precipitation, we performed three atmosphere-only simulations using prescribed SSTs from the corresponding coupled experiments. A full setup detail is provided in Table 1 and Materials and Methods.

In both Atmos_EuraIndAfr and Atmos_EuraIndAfrNA experiments, the precipitation anomalies differ notably from their fully coupled counterparts (fig. S2, A and B). Specifically, summer precipitation has limited changes over the EASM and ISM regions but increases over the ocean south of Asia in the AGCM runs compared to the AOGCMs. This highlights the importance of using a fully coupled AOGCM. Using atmosphere-only GCM with prescribed SSTs and sea ice can remove important nonlinear feedback from the ocean as well as nonrealistic processes in the simplified continental configuration, for example, nonrepresentative ocean circulation and heat transport.

The third experiment, NA_LandOnly, uses the land-sea mask of EuraIndAfrNA but is forced by SSTs from the EuraIndAfr simulation. Comparing this experiment to the other two AGCM runs allows us to separate the effects of North America’s landmass from its SST influence. Specifically, when isolating North America’s continental effects alone (fig. S2C), there are large precipitation increase over both the EASM (1.77 mm/day) and ISM (2.34 mm/day) regions. When including North America–induced SST changes as well (fig. S2D), the additional effect is minor. These results suggest that the North America’s continental effects play a more dominant role than its SST impact in enhancing Asian summer precipitation.

### North American impact in the presence of Tibet

Tibetan orography has seen notable attention in forcing the Asian monsoon (*6*, *11*). Therefore, in our model, we add an idealized Tibet to confirm the robustness of the North American teleconnection and to assess the relative contributions of North America and Tibet on the Asian monsoon system.

**Fig. 4.**
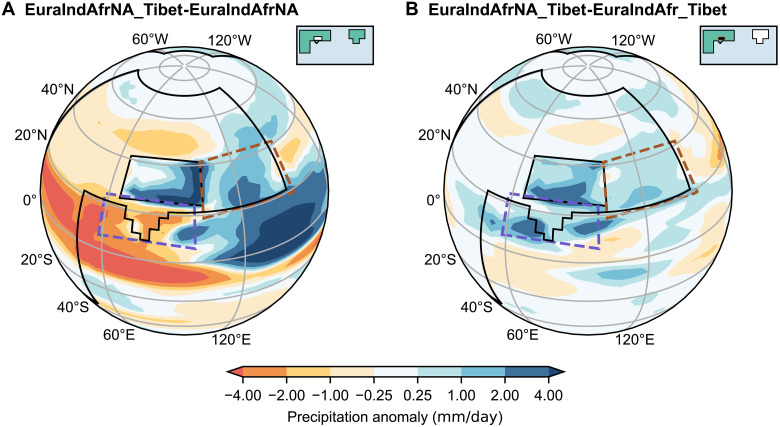
JJAS precipitation anomalies from Tibetan or North American additions. Precipitation anomalies (millimeters per day, shading) show the effects due to (**A**) additional Tibet (EuraIndAfrNA_Tibet-EuraIndAfrNA) and (**B**) additional North America in the presence of Tibet (EuraIndAfrNA_Tibet-EuraIndAfr_Tibet). Inset maps show global land-sea-mountain masks, where white shading indicates the added land or orography.

When Tibet is included, the EASM precipitation increases both with and without North America present (Fig. 4A and fig. S1, T to Y). Monsoon precipitation over EASM region shifts from a basically zonal pattern (fig. S1, F, H, and P) to a southwest-to-northeast pattern (fig. S1, T, V, and X). In contrast, ISM precipitation decreases with the addition of idealized Tibet (Fig. 4A and fig. S1, T to Y).

The positive North America–induced effects that enhance Asian summer monsoon precipitation persist even when Tibet is included, resulting in a total JJAS precipitation increase by around 1.68 mm/day (Fig. 4B). Over the EASM region, the precipitation increase induced by North America is smaller when Tibet is included (0.66 mm/day; Fig. 4B) compared to when Tibet is absent (1.28 mm/day; Fig. 3A). In contrast, over the ISM region, the increase is larger when Tibet is present (1.02 mm/day; Fig. 4B) compared to the flat-continent cases (0.63 mm/day; Fig. 3A).

To compare the relative contributions of North America and the Tibetan Plateau quantitatively, we calculate the percentage change in regional precipitation using the EuraIndAfrNA_Tibet experiment as a reference. The North American contribution is estimated by removing North America from this setup and, similarly, the Tibetan contribution by removing Tibet. Full equations and details are provided in the Supplementary Materials.

The results reveal that, over the EASM region, Tibet contributes a 34% increase in summer precipitation (Fig. 4A), whereas North America contributes a 17% increase (Fig. 4B). Hence, Tibet is the largest driver, but North America is half as important. In contrast, over the ISM region, Tibet causes a 41% decrease (Fig. 4A), whereas North America drives a 28% increase (Fig. 4B). Thus, North America is more crucial than our idealized Tibet for stronger ISM precipitation.

## DISCUSSION

### Mechanisms of North American effects

**Fig. 5.**
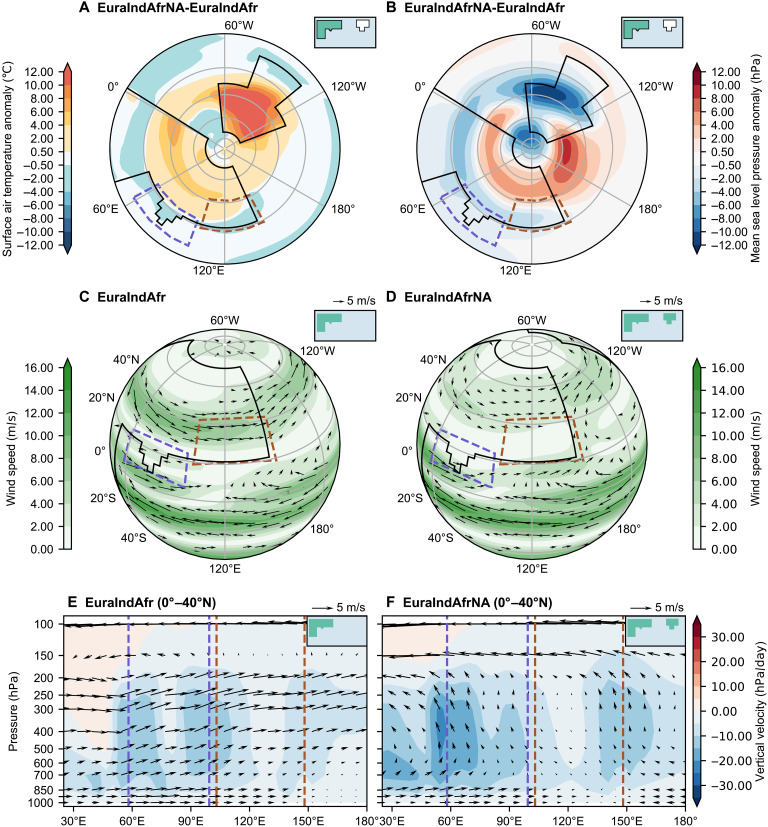
JJAS thermodynamic response to North American forcing. (**A**) Surface air temperature anomaly (degrees Celsius, shading) between EuraIndAfrNA and EuraIndAfr. (**B**) Mean sea level pressure anomaly (hectopascals, shading) between EuraIndAfrNA and EuraIndAfr. (**C** and **D**) Wind speed (meters per second, shading) and horizontal wind vectors at 850 hPa (meters per second, arrows; only wind speeds >2 m/s shown) for (C) EuraIndAfr and (D) EuraIndAfrNA. (**E** and **F**) Zonal mean vertical velocity (hectopascals per day, shading) averaged over 0° to 40°N, overlaid with zonal and vertical wind vectors (meters per second, arrows; vertical wind vectors scaled by −500 for visualization) for (E) EuraIndAfr and (F) EuraIndAfrNA. In [(E) and (F)], brown dashed lines mark the longitudinal extent of the EASM region; purple dashed lines mark that of ISM region.

The main role of North America is acting as an additional heating center to Eurasia in the zonal direction. The basic mechanism parallels to that seen in the study of Farnsworth *et al.* (*34*), who saw a reduction in East Asian monsoon when the Western Interior Seaway in North America was open during the late Cretaceous. We see the reverse process, whereby a change from ocean to land results in strong warming during the boreal summer over North America, which extends throughout the mid-latitudes (Fig. 5A). The warming and related enhanced diabatic heating (fig. S3, A and B) result in low pressure over North America and a stronger eastern North Pacific subtropical high-pressure system (Fig. 5B). This mechanism is similar to that described by Rodwell and Hoskins (*35*), in which localized diabatic enhancement forces a Rossby wave response that expands westward and interacts with midlatitude westerlies, inducing a region of enhanced descent to the west of the heating region. The associated surface warming across the Northern Hemisphere also widens the Hadley circulation whereby the descending center and the midlatitude westerlies shift poleward (Fig. 5, C and D, and fig. S3, C to E), as described by D’Agostino *et al.* (*36*). The strengthened North Pacific subtropical high, along with a negative pressure anomaly over southern Eurasia (Fig. 5B), increases the surface pressure gradient between the Asian monsoon region and the surrounding oceans, leading to the intensified low-level jets (Fig. 5, C and D). These circulation changes promote stronger moisture convergence toward Asia from the south and southeast (Fig. 5, C and D) and excite deeper convection over the Asian monsoon region (Fig. 5, E and F, and fig. S3, C and D). The relationship between North Pacific subtropical high pressure system and East Asian summer precipitation was found to be a robust feature of various climate models (*37*).

Additional AGCM experiments that isolate atmospheric and oceanic effects reveal that North America’s continental effects on Asian summer precipitation are more pronounced than those induced by remote SST anomalies. This finding is supported by Zhao *et al.* (*17*), who found that land surface heating over the Eurasian non-monsoon region and North America is the primary driver of Asian summer precipitation anomalies, whereas El Niño–Southern Oscillation–related ocean forcing is secondary and tends to be confined to the tropics. Our findings might be explained by the spatial heterogeneity of SST anomaly pattern. In our simulations, SST anomalies induced by the addition of North America are strongest over the North Pacific and North Atlantic but are relatively weak near southern and southeastern Asia (fig. S3F), where monsoon responses are most sensitive. This spatial mismatch likely limits their impact. Besides, a previous study has shown that positive SST anomaly in the Atlantic and the associated strengthened Atlantic Meridional Overturning Circulation (AMOC) have no obvious impact on global monsoon annual mean precipitation, unlike the AMOC weakening scenarios (*38*).

### Mechanisms combined North American and Tibetan effects

**Fig. 6.**
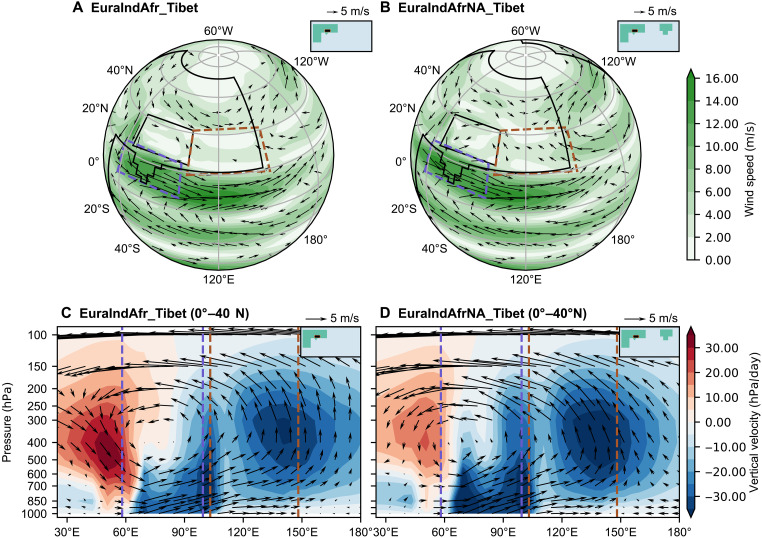
JJAS thermodynamic response to combined North American and Tibetan forcing. Same variables as in Fig. 5 (C to F) but for (**A** and **C**) EuraIndAfr_Tibet and (**B** and **D**) EuraIndAfrNA_Tibet.

The role of Tibetan orography on EASM and ISM precipitation has long been a hot topic and is still under debate (*6*). In our experiments, we show a precipitation increase in the EASM due to the inclusion of Tibet, which is consistent with many previous studies (*10*, *23*, *39*, *40*), while a precipitation decrease to ISM (Fig. 4A and fig. S1, U and W). Some related thermodynamic changes around the ISM region can also be seen, such as strong northwesterly flow along the southwestern flank of the Tibet (Fig. 6, A and B, and fig. S1, T and V), cooler SSTs around India (fig. S4A), and broad atmospheric subsidence over the northern India (fig. S4, C and D).

This is because the Tibet in our model is represented as a square block with a uniform elevation of ~4500 m and a western boundary only at 67.5°E, without any other peripheral orography, such as the Himalayas, the Iranian Plateau, and the East African highlands. This surrounding orography is also important or even more crucial than the Tibetan Plateau itself in shaping the modern Asian monsoon, especially the pronounced ISM precipitation (*40*–*44*). The suppressed ISM precipitation seen in our pure-Tibet configuration is consistent with other studies that isolate Tibet from surrounding highlands (*10*, *40*, *41*, *43*), supporting the robustness of our results. Some studies reporting increased ISM precipitation in response to Tibetan uplift [e.g., (*13*)] often raise surrounding terrain as well, making them less directly comparable to Tibet-only experiments.

The North American contribution to Asian summer precipitation enhancement remains robust, even in the presence of Tibet. When Tibet is included, it strengthens the “North Pacific” subtropical high (fig. S4B) and low-latitude easterlies (Fig. 6, A and B). Adding North America further reinforces the subtropical high (fig. S4F) and widens the Northern Hemispheric Hadley cell (fig. S6E), all of which account for a further enhancement of convective activity and large-scale ascent around India and southeastern Asia (Fig. 6, C and D, and fig. S4, C and D). This enhances the positive Tibetan effects on EASM precipitation and partially offsets the negative effects on ISM precipitation.

For EASM precipitation, the Tibetan Plateau emerges as the dominant driver, with North America playing a secondary role. This relative influence can be explained in two ways. First, the wind anomalies over the EASM region and subtropical North Pacific are stronger in response to Tibetan orography (fig. S5A) than to North American teleconnection (fig. S5B). Second, Tibet induces a larger northward shift of the Hadley descendent than that by North America. The latitude of maximum eddy momentum flux shifts northward by 5° with the addition of Tibet (from 45.0°N of EuraIndAfrNA in fig. S3E to 50.0°N of EuraIndAfrNA_Tibet in fig. S4E), compared to a 2.5° shift induced by North America (from 47.5°N of EuraIndAfr_Tibet to 50.0°N of EuraIndAfrNA_Tibet in fig. S4E).

In summary, we have shown that the North American continent plays a substantial role in shaping the strength of the modern Asian monsoon. For the EASM precipitation, the increase due to North America is around half that from the addition of Tibet, and for the ISM precipitation, it reverses the sign of the response induced by Tibet. The mechanism operates via large-scale teleconnections, similar to the Rodwell-Hoskins mechanism.

Our results also emphasize the importance of simulating both a dynamic atmosphere and ocean. Using prescribed SSTs in AGCMs may miss out key processes that are crucial for controlling Asian summer precipitation.

These findings have implications for understanding the geologic evolution of the Asian monsoon system. Throughout geological history, land-sea distribution of Earth has continually shifted. North and South American continents rifted westward from Afro-Eurasia since the early Cretaceous (~143 to 100 Ma) and are projected to converge along the eastern Pacific margin in the future (*45*). Although the land-sea-mountain boundary settings of this study are highly idealized and based on modern geography, CO_2_ levels, etc., it serves as a conceptual starting point for understanding the role of North American continent in Asian monsoon evolution throughout palaeogeographical history and into the long-term future.

This work also has implications for 21st-century predictions of monsoon duration, intensity, and variability. It underscores the importance of hemispheric teleconnections and hence suggests that changes over North America as a result of anthropogenic climate change could also affect the Asian monsoon system.

There are limitations to our experimental design and interpretation. The use of idealized continents, subgrid-scale orography, soil properties, and generalized vegetation within the dynamic vegetation model may limit direct comparability with observations or comprehensive Earth system models. Using more realistic coastlines and topography, including other mountain ranges, subcontinents (e.g., the Indochina Peninsula) and polar ice sheets would yield more insights. On the interpretive side, our analysis primarily emphasizes the mean state of the Asian summer monsoon. Further research is needed to address the winter monsoon, as well as monsoon timing, duration and seasonality, and associated impacts on vegetation and ecosystems.

Last, there are large uncertainties regarding model performance. While we use a state-of-the-art general circulation model with performance comparable to or better than many models in Coupled Model Intercomparison Project Phase 5 (CMIP5) in simulating the modern Asian monsoon (*46*), further investigation using other AOGCMs is necessary to confirm the robustness and generality of our findings, ensuring they are not model dependent.

## MATERIALS AND METHODS

### Model settings

The model adopted is the HadCM3BL, developed by the Bristol Research Initiative for the Dynamic Global Environment research group at the University of Bristol, UK (*47*, *48*). The model has an atmospheric resolution of 3.75° longitude × 2.5° latitude × 19 vertical levels (unequally spaced, giving more resolution at the surface and tropopause) and an ocean resolution of 3.75° × 2.5° × 20 vertical levels (unequally spaced with more resolution near the surface, with a total depth near to 6000 m). The ocean model requires land at both poles to avoid singularities. In the modern geographical configuration, this requires an artificial “polar island” at the North Pole only, but in some of our revised continent distributions, we require a polar island at the South Pole as well.

The model includes a normal range of atmospheric and ocean parameterizations. It also includes the MOSES3.1a land surface exchange scheme which allows coupling to a dynamic vegetation module (Top-down Representation of Interactive Foliage and Flora Including Dynamics, TRIFFID). For a fuller description, see the study of Valdes *et al.* (*47*) for the specific setup of HadCM3BL-M2.1aD. The model performance in simulating the modern Asian monsoon characteristics is comparable to or exceeds that of many CMIP5 generation models (*46*).

To conduct idealized simulations, some important modifications to the standard models are made. A weak quadratic ocean bottom friction parameterization is used to account for turbulence and tidal effects. In addition, the seafloor is flat except for two bottom ridges to further increase ocean bottom friction to avoid super rotation. The ridges are 3500 to 4500 m in height, located at 72.5° to 90°N, 71.25° to 78.75°E and 75° to 90°N, 90° to 97.5°W, respectively. Solar forcing and all trace gases are set to PI values, e.g., 280 ppmv for CO_2_. All simulations begin from the same zonal mean initial conditions derived from a PI control run. Each simulation is integrated for 4500 model years to ensure near equilibrium in the atmosphere and deep ocean, removing the influence of initial conditions. Climatological means are calculated from the last 100 years of each simulation to ensure interannual/interdecadal variability influence on the monsoon signal is removed.

### Sensitivity experiments

This study uses the ALL_Tibet experiment as the reference configuration, which features all idealized flat continents and a simplified, block-like representation of the Tibetan Plateau. The idealized Tibet extends from 25.00° to 40.00°N and 67.50° to 101.25°E, with a uniform elevation of 4500 m and marginal slopes to smooth transitions between grid cells. To assess the robustness of the results, we conduct the Realistic_PI experiment, which incorporates modern continental geometry and topography, with PI atmospheric CO_2_ concentrations. In addition, we also include observational data from Global Precipitation Climatology Project for year 1981–2010, regridded to match the model resolution.

For the sensitivity experiments (Table 1), we start from an aquaplanet entirely covered by ocean water. Landmasses with highly simplified coastlines and flat topography are added sequentially, as separate snapshot simulations rather than a single continuous transient simulation, to represent modern continents. These include Eurasia, India, and Africa, followed by separate additions of flat Australia, Antarctica, North America, and South America. When both Americas are present, there is no flow between the idealized Panama isthmus.

Additional experiments include one with all continents (ALL) and one combining all continents but excluding North America (ALL-noNA). The setting of these experiments aims to consider the interactions among continents. In some of the previous studies, the idealized Eurasia continent extends to 90°N (*9*, *10*). Therefore, we also create an expanded Eurasia with similar meridional extension for comparison, which show little difference of JJAS precipitation between the normal (fig. S1B) and extended Eurasia experiments (fig. S1, Z and AA).

To isolate atmospheric and oceanic contributions, we conduct AGCM experiments using HadAM3BL, the atmospheric component of HadCM3BL. Each simulation is forced with prescribed monthly SST and sea ice fields derived from the last 100 years of the corresponding fully coupled AOGCM experiments. Atmos_EuraIndAfr uses the land-sea mask and SSTs from the EuraIndAfr run. Atmos_EuraIndAfrNA follows the same protocol, using the land-sea mask and SSTs from EuraIndAfrNA. NA_LandOnly uses the land-sea mask of EuraIndAfrNA but with SSTs from the EuraIndAfr run, allowing isolation of the land-only effect of North America. All AGCM experiments are integrated for 100 years after a 300-year spin-up.
