# Supplementary material for: The role of the North American continent in strengthening the Asian summer monsoon
Source: Sci Adv. 2025 Sep 5;11(36):eadu8589. doi: 10.1126/sciadv.adu8589 (PMC12412649; doi:10.1126/sciadv.adu8589)
Supplement: Supplementary file 1 — Supplementary Text Figs. S1 to S5 Tables S1 and S2 [file sciadv.adu8589_sm.pdf]

Science Advances
AAAS

Supplementary Materials for

The role of the North American continent in strengthening the Asian summer monsoon

Linlin Chen *et al.*

Corresponding author: Linlin Chen, linlin.chen@bristol.ac.uk

Sci. Adv. **11**, eadu8589 (2025)
DOI: 10.1126/sciadv.adu8589

This PDF file includes:

Supplementary Text
Figs. S1 to S5
Tables S1 and S2

Supplementary Text: Calculation of North American and Tibetan contributions

The fractional contributions of North America and Tibet to precipitation in each target region are calculated using the following equations:

$$C_{NA,region} = \frac{P_{EuraIndAfrNA\_Tibet} - P_{EuraIndAfr\_Tibet}}{P_{EuraIndAfrNA\_Tibet}} \times 100\% \quad (1)$$

$$C_{Tibet,region} = \frac{P_{EuraIndAfrNA\_Tibet} - P_{EuraIndAfrNA}}{P_{EuraIndAfrNA\_Tibet}} \times 100\% \quad (2)$$

Where:

- $C_{NA,region}$ and $C_{Tibet,region}$ represent the percentage contributions of North America and Tibet, respectively, to precipitation in a given target region.
- P_x denotes the precipitation (mm/day) from experiment x, averaged over the target region.

Using Equation (1), the contributions of North America to JJAS precipitation are:

- EASM region:

$$C_{NA,EASM} = \frac{3.93 - 3.27}{3.93} \times 100\% = 16.79\%$$

- ISM region:

$$C_{NA,ISM} = \frac{3.65 - 2.63}{3.65} \times 100\% = 27.95\%$$

Using Equation (2), the contributions of Tibet to JJAS precipitation are:

- EASM region:

$$C_{Tibet,EASM} = \frac{3.93 - 2.58}{3.93} \times 100\% = 34.35\%$$

- ISM region:

$$C_{Tibet,ISM} = \frac{3.65 - 5.14}{3.65} \times 100\% = -40.82\%$$

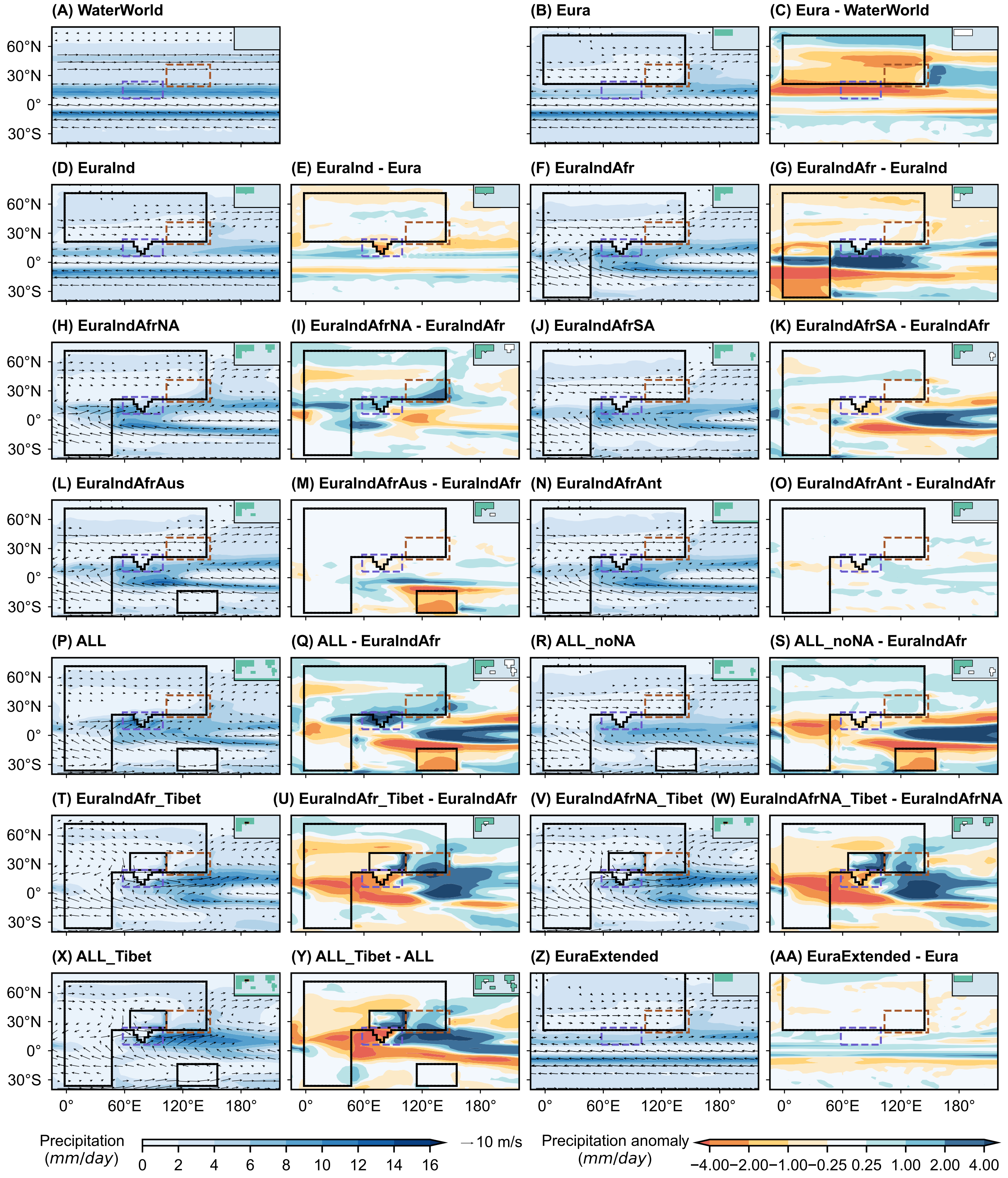

Fig. S1. JJAS precipitation and 850 hPa winds for all experiments. First and Third columns show precipitation (mm/day, shading) overlaid with horizontal wind vectors at 850 hPa (m/s, arrows; only wind speeds > 2 m/s shown). Second and Fourth columns show precipitation anomalies (mm/day, shading). Black lines indicate land-sea-mountain boundaries. Inset maps show global land-sea-mountain masks, with white shading indicating added land and/or orography. Brown and purple dashed boxes mark the EASM and ISM regions, respectively.

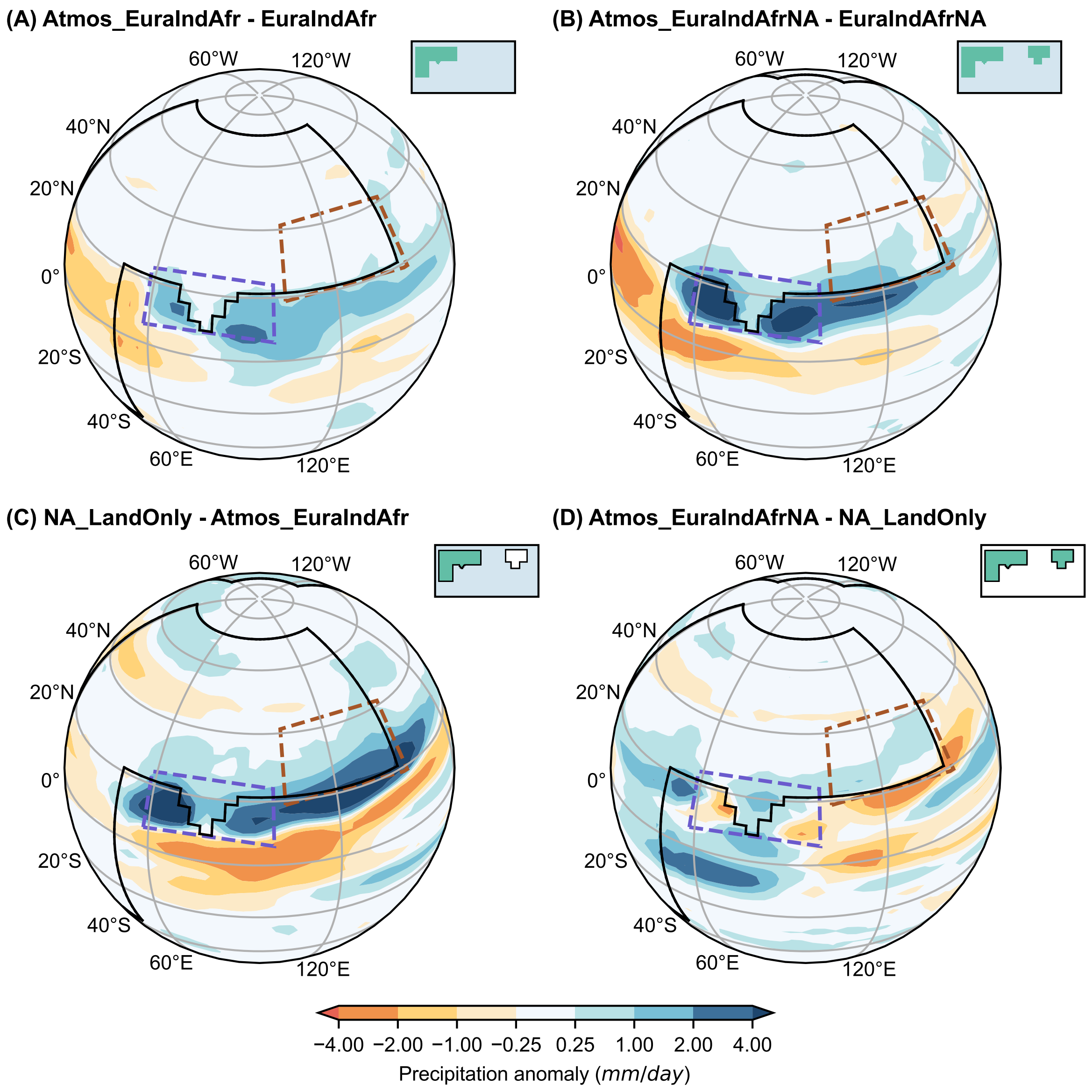

Fig. S2. Atmospheric and oceanic contributions to North American forcing. JJAS precipitation anomalies (mm/day, shading) between (**A**) Atmos_EuraIndAfr and EuraIndAfr, (**B**) Atmos_EuraIndAfrNA and EuraIndAfrNA, (**C**) NA_LandOnly and Atmos_EuraIndAfr, isolating the effect of adding North American landmass while prescribing SSTs from EuraIndAfr (continental effect), and (**D**) Atmos_EuraIndAfrNA and NA_LandOnly, isolating the effect of North America-induced SST changes added to the continental effect (SST effect). Inset maps show global land-sea-mountain masks, with white shading indicating the land or SST changes in each case.

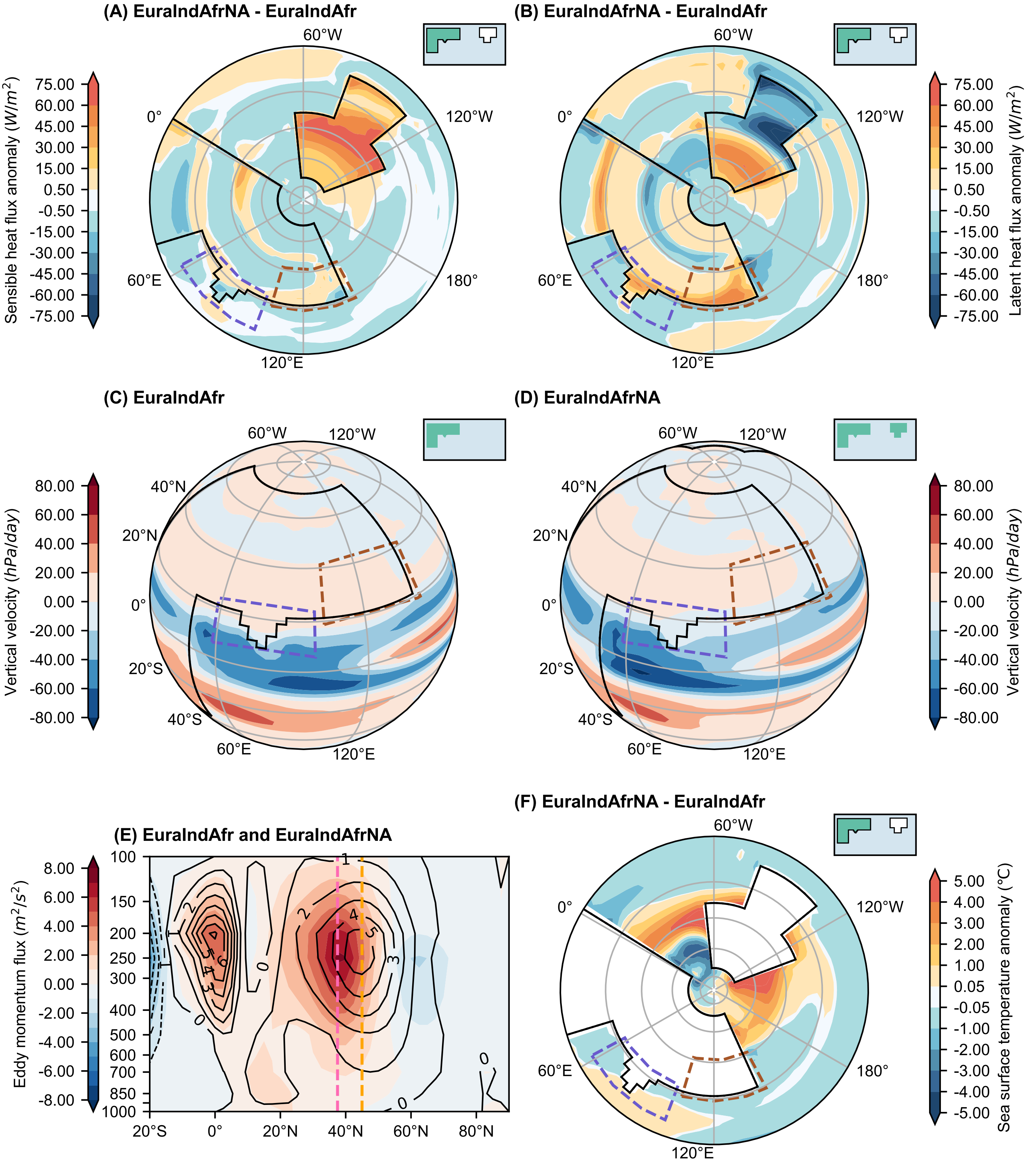

Fig. S3. Additional diagnostics of the JJAS thermodynamic response to North American forcing. (**A**) Sensible heat flux anomaly (W/m^2, shading) and (**B**) Latent heat flux anomaly (W/m^2, shading) between EuraIndAfrNA and EuraIndAfr. Vertical velocity at 500 hPa (hPa/day, shading) for (**C**) EuraIndAfr and (**D**) EuraIndAfrNA. (**E**) Zonal mean of eddy momentum flux (m^2/s^2) for EuraIndAfr (shading) and EuraIndAfrNA (black contours). Positive values indicate northward transport of eastward momentum, and negative values indicate southward transport of eastward momentum. Dashed line highlights the latitude of maximum flux in the Northern Hemisphere: 37.5°N for EuraIndAfr (pink), and 45.0°N for EuraIndAfrNA (orange). (**F**) Sea surface temperature anomaly (°C, shading) between EuraIndAfrNA and EuraIndAfr.

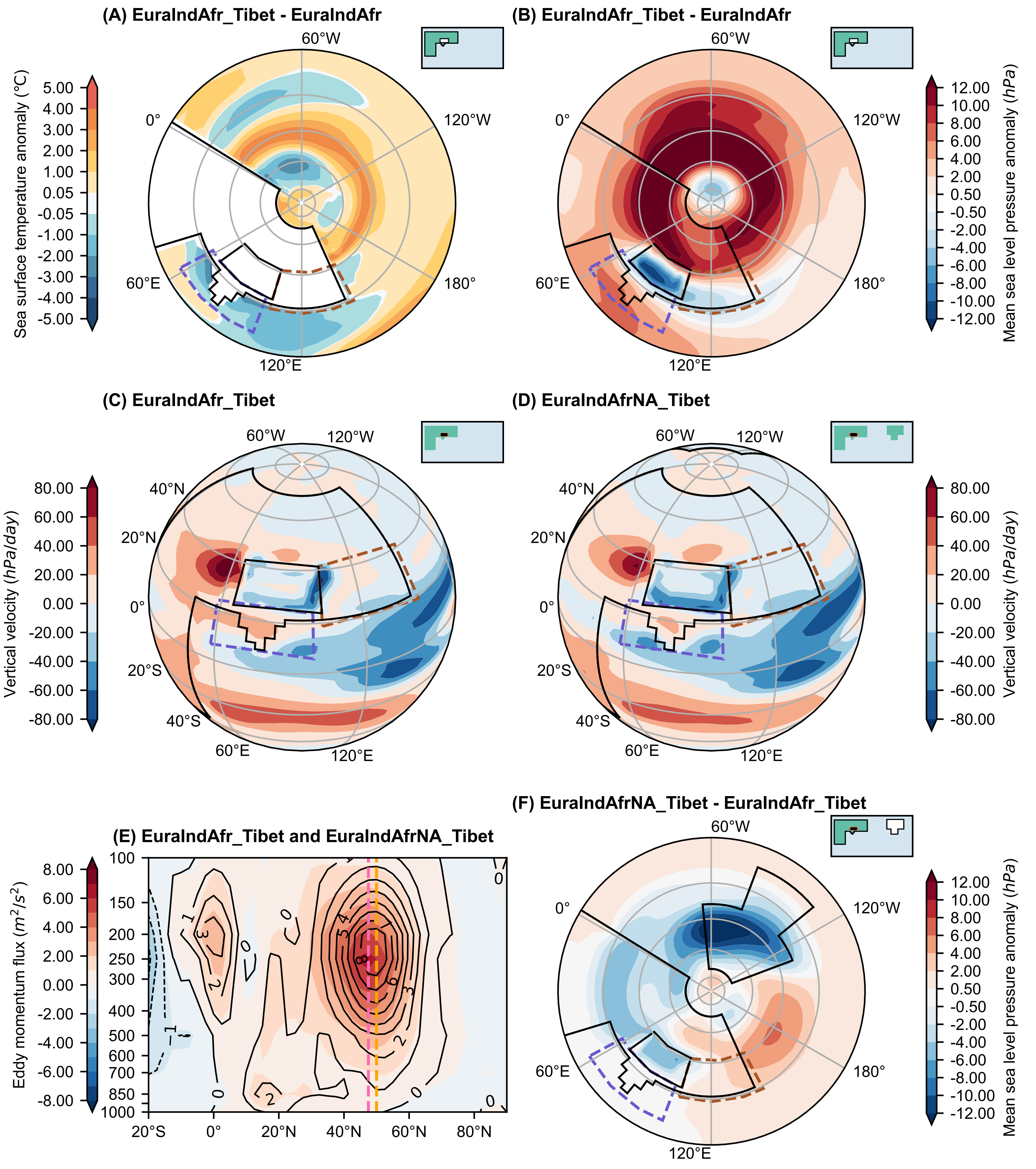

Fig. S4. Additional diagnostics of the JJAS thermodynamic response to combined North American and Tibetan forcing. (**A**,**B**) Anomalies between EuraIndAfr_Tibet and EuraIndAfr due to additional Tibet: (**A**) Sea surface temperature anomaly (°C, shading) and (**B**) Mean sea level pressure anomaly (hPa). (**C**–**F**) Anomalies due to additional North America in the presence of Tibet: vertical velocity at 500 hPa (hPa/day, shading) for (**C**) EuraIndAfr_Tibet and (**D**) EuraIndAfrNA_Tibet. (**E**) Zonal mean of eddy momentum flux (m^2/s^2) for EuraIndAfr_Tibet (shading) and EuraIndAfrNA_Tibet (black contours). Dashed line highlights the latitude of maximum flux in the Northern Hemisphere: 47.5°N for EuraIndAfr_Tibet (pink) and 50.0°N for EuraIndAfrNA_Tibet (orange). (**F**) Mean sea level pressure anomaly (hPa, shading) between EuraIndAfrNA_Tibet and EuraIndAfr_Tibet.

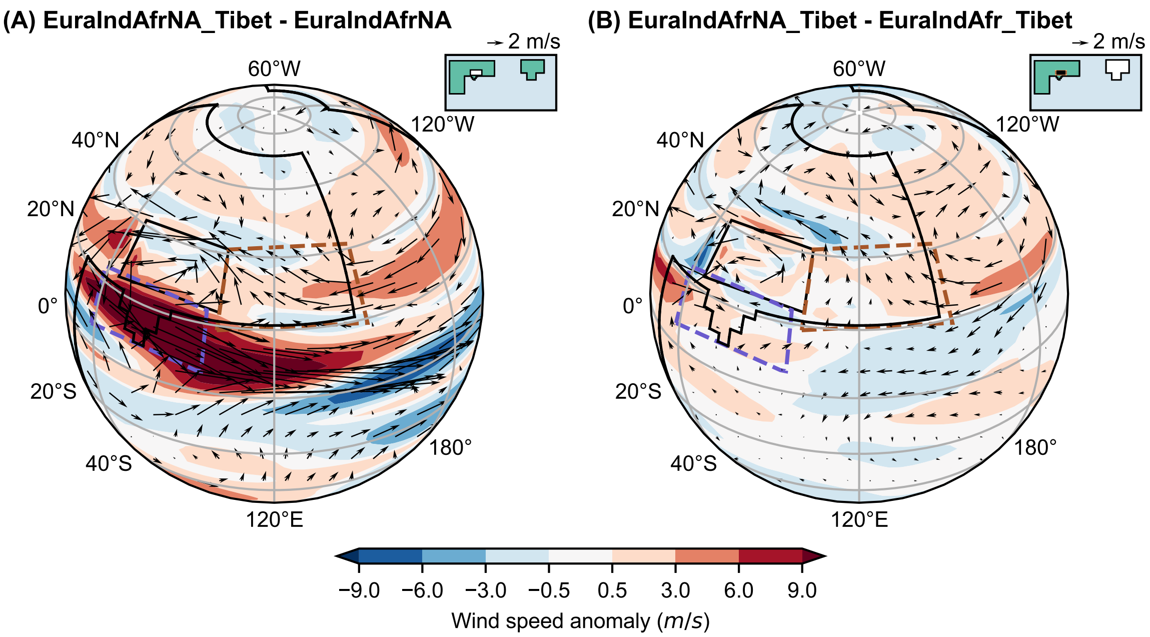

Fig. S5. Comparison of Tibetan and North American effects. JJAS wind speed anomalies (m/s, shading) and horizontal wind vector anomalies (m/s, vector) at 850 hPa showing the effects due to (**A**) additional Tibet (EuraIndAfrNA_Tibet - EuraIndAfrNA) and (**B**) additional North America in the presence of Tibet (EuraIndAfrNA_Tibet - EuraIndAfr_Tibet).

Table S1. Geographical boundaries of simplified continental configurations.

Continent	Geographical boundaries [a)]
Eurasia	22.50°–70.00°N, 0.00°–142.50°E
India	20.00°N, 71.25°–86.25°E; 17.50°N, 71.25°–82.5°E; 15.00°N, 75.00°–82.50°E; 12.50°N, 75.00°–78.75°E; 10.00°N, 78.75°E
Africa	35.00°S–20.00°N, 0.00°–45.00°E
Australia	15.00°–35.00°S, 116.25°–153.75°E
Antarctica	72.50°–90.00°S, 0.00°–360.00°
North America	32.50°–72.50°N, 56.25°–127.50°W; 10.00°–30.00°N, 82.50°–108.75°W
South America	0.00°–10.00°N, 52.50°–75.00°W; 2.50°–25.00°S, 37.50°–78.75°W; 27.50°–52.50°S, 56.25°–71.25°W
Extended Eurasia	22.50°–90.00°N, 0.00°–142.50°E

[a)] Coordinates indicate the center of each model grid cell, with a longitudinal resolution of 3.75° and a latitudinal resolution of 2.5°.

Table S2. JJAS precipitation anomalies across experiments.

		EASM region				ISM region				EASM and ISM region			
Job1	Job2	Job1 (mm/day)	Job2 (mm/day)	Anomaly (Job2 - Job1, mm/day)	Anomaly Percent relative to ALL_Tibet (%) [a)]	Job1 (mm/day)	Job2 (mm/day)	Anomaly (Job2 - Job1, mm/day)	Anomaly Percent relative to ALL_Tibet (%) [b)]	Job1 (mm/day)	Job2 (mm/day)	Anomaly (Job2 - Job1, mm/day)	Anomaly Percent relative to ALL_Tibet (%) [c)]
WaterWorld	Eura	3.09	1.73	−1.36	−35	7.35	4.18	−3.17	−70	10.44	5.91	−4.53	−54
Eura	EuraInd	1.73	1.59	−0.14	−4	4.18	3.59	−0.59	−13	5.91	5.18	−0.73	−9
EuraInd	EuraIndAfr	1.59	1.30	−0.29	−7	3.59	4.51	0.92	20	5.18	5.81	0.63	7
EuraIndAfr	EuraIndAfrAus	1.30	1.24	−0.06	−2	4.51	4.27	−0.24	−5	5.81	5.51	−0.30	−4
EuraIndAfr	EuraIndAfrAnt	1.30	1.35	0.05	1	4.51	4.49	−0.02	0	5.81	5.84	0.03	0
EuraIndAfr	EuraIndAfrNA	1.30	2.58	1.28	32	4.51	5.14	0.63	14	5.81	7.72	1.91	23
EuraIndAfr	EuraIndAfrSA	1.30	1.21	−0.09	−2	4.51	4.01	−0.50	−11	5.81	5.22	−0.59	−7
EuraIndAfr	ALL	1.30	2.43	1.13	29	4.51	7.08	2.57	57	5.81	9.51	3.70	44
EuraIndAfr	ALL_noNA	1.30	1.50	0.20	5	4.51	3.98	−0.53	−12	5.81	5.48	−0.33	−4
EuraIndAfr	EuraIndAfr_Tibet	1.30	3.27	1.97	50	4.51	2.63	−1.88	−42	5.81	5.90	0.09	1
EuraIndAfrNA	EuraIndAfrNA_Tibet	2.58	3.93	1.35	34	5.14	3.65	−1.49	−33	7.72	7.58	−0.14	−2
EuraIndAfr_Tibet	EuraIndAfrNA_Tibet	3.27	3.93	0.66	17	2.63	3.65	1.02	23	5.90	7.58	1.68	20
ALL	ALL_Tibet	2.43	3.94	1.51	38	7.08	4.52	−2.56	−57	9.51	8.46	−1.05	−12
Eura	EuraExtend	1.73	1.68	−0.05	−1	4.18	4.10	−0.08	−2	5.91	5.78	−0.13	−2
EuraIndAfr	Atmos-EuraIndAfr	1.30	1.50	0.20	5	4.51	5.48	0.97	21	5.81	6.98	1.17	14
EuraIndAfrNA	Atmos-EuraIndAfrNA	2.58	3.13	0.55	14	5.14	7.95	2.81	62	7.72	11.08	3.36	40
Atmos-EuraIndAfr	NA_LandOnly	1.50	3.27	1.77	45	5.48	7.82	2.34	52	6.98	11.09	4.11	49
NA_Land Only	Atmos-EuraIndAfrNA	3.27	3.13	−0.14	−4	7.82	7.95	0.13	3	11.09	11.08	−0.01	0

[a)] $Anomaly\ Percent\ relative\ to\ ALL\_Tibet\ (\%)_{EASM} = Anomaly_{Job2-Job1,EASM}/Precipitation_{ALL\_Tibet,EASM} \times 100 = Anomaly_{Job2-Job1,EASM}/3.94 \times 100$

[b)] $Anomaly\ Percent\ relative\ to\ ALL\_Tibet\ (\%)_{ISM} = Anomaly_{Job2-Job1,ISM}/Precipitation_{ALL\_Tibet,ISM} \times 100 = Anomaly_{Job2-Job1,ISM}/4.52 \times 100$

[c)] $Anomaly\ Percent\ relative\ to\ ALL\_Tibet\ (\%)_{EASM\&ISM} = Anomaly_{Job2-Job1,EASM\&ISM}/Precipitation_{ALL\_Tibet,EASM\&ISM} \times 100 = Anomaly_{Job2-Job1,EASM\&ISM}/8.46 \times 100$
